# Combining high carotenoid, grain protein content and rust resistance in wheat for food and nutritional security

**DOI:** 10.3389/fgene.2023.1075767

**Published:** 2023-01-19

**Authors:** Asish Kumar Padhy, Achla Sharma, Himanshu Sharma, Puja Srivastava, Satinder Singh, Parampreet Kaur, Jaspal Kaur, Satinder Kaur, Parveen Chhuneja, Navtej Singh Bains

**Affiliations:** Punjab Agricultural University, Ludhiana, India

**Keywords:** bread wheat (*Triticum aestivum* L), biofortifcation, carotenoids, protein, *GpcB1* gene, *PsyE1* gene

## Abstract

Globally, malnutrition has given birth to an alarming predicament, especially in developing countries, and has extensively shifted consumer preferences from conventional high-energy diets to a nutritionally balanced, cost-effective, sustainable, and healthy lifestyle. In keeping with this view and the mandate for developing high-yielding, disease-resistant biofortified staple food (wheat) for catering to the demand-driven market, the current research aimed at stacking together the enhanced grain protein content, carotenoid content, and disease resistance in an elite bread wheat background. The *Y* gene (*PsyE1*) and the *GpcB1* gene were used as novel sources for enhancing the grain carotenoid and protein content in the commercial elite bread wheat cultivar HD2967. The combination also led to the stacking of resistance against all three foliar rusts owing to linked resistance genes. A stepwise hybridization using Parent 1 (HD2967 + *PsyE1/Lr19/Sr25*) with Parent 2 (PBW550 + *GpcB1/Yr36+ Yr15*), coupled with a phenotypic-biochemical selection, narrowed down 2748 F_2_ individuals to a subset of 649 F_2_ plants for molecular screening. The gene-specific markers *PsyE1, PsyD1, Xucw108*, and *Xbarc8* for the genes *PsyE1, PsyD1, GpcB1,* and *Yr15,* respectively, were employed for forward selection. Four bread wheat lines positive for all the desired genes with high carotenoid (>8ppm) and protein (>13%) content were raised to the F_5_ generation and will be evaluated for yield potential after bulking. These improved advanced breeding lines developed following multipronged efforts should prove a valuable and unique source for the development of cultivars with improved nutritional quality and rust resistance in wheat breeding programs.

## Highlights

Combining unique nutritional subcomponents (high carotenoid and protein content in wheat grain) along with rust resistance for food as well as nutritional security using conventional and marker-assisted breeding.

## 1 Introduction

Wheat (*Triticum aestivum* L.) is one of the major staples consumed by approximately 2.5 billion people globally to meet the 20% of dietary calorie value (Velu et al., 2017). Wheat is utilized in various forms such as bread, *chapati*, semolina, pasta, macaroni, noodles, and biscuits. with an annual *per capita* consumption of 67.4 kg (Padhy et al., 2022b). On the other hand, FAO (Food and Agriculture Organization) reported that two billion people from developing countries suffer from “hidden hunger” due to inadequate intake of essential micronutrients (Garg et al., 2018). Wheat biofortification with essential nutrients offers a long-term and sustainable approach to nutritional security. Wheat being the staple diet, the envisaged product will have greater reachability to the masses, including the low-income groups with low affordability to costly additional supplements. Further, consumer awareness of a healthy lifestyle in the past decade has played a pivotal role in revamping food. Following the trend and market demand, several biofortified wheat varieties for iron, zinc, selenium, anthocyanins, etc., are now available commercially (Garg et al., 2018). Recently, biofortified colored wheat has drawn significant attention among manufacturers, food processors, and consumers due to its potential as a food colorant, nutraceutical ingredient, and prospective functional food (Sharma et al., 2018; Padhy et al., 2022b). Pigments, particularly anthocyanins, flavonoids, phlobaphenes, and catechins responsible for black, blue, purple, and other colors of colored wheat and their products are generally localized in the outer layers (aleurone/pericarp) of grains, and thereby facilitate their commercial extraction (Lachman et al., 2017), while yellow-colored carotenoids are housed in the grain endosperm (Clydesdale, 1993; Mezzomo, and Ferreira, 2016).

Traditionally, white or amber-colored *chapati* (Indian flat bread) was preferred whereas yellow-colored flour was utilized to prepare biscuits, pasta, semolina, and alkaline noodles, consumed in Japan and South-East Asia (Kadam et al., 2012; Padhy et al., 2022b). However, blended *chapatis,* commonly known as *missi roti,* (made from wheat and chickpea flour in a ratio of 80:20, Kadam et al., 2012; Dhillon et al., 2022) are light yellowish in color and is one of the most relished forms of *chapati* in Northern India. Usually, to cut down the costs, these are prepared commercially (in restaurants, functions, etc.) using food colorants to give a yellowish color, thus lowering their nutritive value (Kadam et al., 2012; Padhy et al., 2022b). Carotenoid biofortification in bread wheat, which is still an underutilized and less explored facet, can be best employed in the above context to meet consumer demands and thus, replace the harmful food colorants. Commercially available bread wheat varieties are generally low in carotenoid content, which ranges from 0.1–3.0 ppm (Hidalgo et al., 2006). However, einkorn wheat (*T. monococcum*) and wild wheat grass (*Thinopyrum* spp., especially, *Th. elongatom* and *Th. ponticum*) have been reported to contain a high amount of carotenoids with a range of 5.3–13.6 ppm (Abdel-Aal et al., 2002; Padhy et al., 2022a; Padhy A. K. et al., 2022), thereby opening avenues for carotenoid biofortification in bread wheat. These genes, therefore, if pyramided with other nutritional and agriculturally important attributes, could potentially translate wheat into a complete staple food with higher nutritive value and health benefits.

The current study was undertaken to combine high carotenoid and grain protein content in bread wheat through the pyramiding of the *PsyE1* (*Y* gene) and *GpcB1* genes. *GpcB1*as a choice for gene pyramiding is unique as it is reported to accentuate grain zinc and iron content (Brevis and Dubcovsky, 2010) and is also linked to *Yr36,* an effective high-temperature adult-plant (HTAP) stripe rust resistance gene (Uauy et al., 2005). Also, the gene for high carotenoid content, *PsyE1*(*Y* gene), from *Th. ponticum* background, is linked to genes *Lr19* and *Sr25*, and both genes have demonstrated efficacy in controlling leaf and stem rust, respectively (Knott, 1989; Zhang et al., 2005). *Sr25* has also been found to be effective against the deadly Ug99 race of stem rust pathogen (Liu and Hambleton, 2010). The choice of genes thus incorporates rust resistance in the envisaged product as an added benefit, considering the fact that rusts in wheat are among the major limiting factors contributing to significant yield losses (Braun et al., 2010). The manuscript reports the pyramiding of *Y* gene (*PsyE1*), *GpcB1,* and stripe rust resistance gene *Yr15* for nutritionally enhanced quality wheat with resistance to all the three foliar rusts, utilizing ‘transfer first and assemble later’ technique in elite bread wheat variety, HD2967.

## 2 Material and methods

### 2.1 Plant materials

Two germplasm sets were used for crossing and generating segregating population for combining *PsyE1* (*Y* gene) and *GpcB1* gene. First set of germplasm consisted of BC_1_F_5_bulk plants having*PsyE1* (*Y* gene) in HD2967 background (HD2967 + *PsyE1/Sr25/Lr19*). The set consisted of 180 lines, out of which an agronomically better-performing line with high pigment content was chosen and used as parent 1 (Padhy et al., 2022c). A commercial bread wheat variety HD2967 (ALD/COC//URES/3/HD2160 M/HD2278) developed by the Indian Agricultural Research Institute (IARI), New Delhi for cultivation under timely sown irrigated conditions in the northwestern plain zone of India (notification number 2326 (E) dated 10.10.2011, Indian Council of Agricultural Research (ICAR), Government of India). The variety has an average yield of 5.1 t/ha (potential yield 6.6 t/ha) with bold, round, lustrous, and amber-colored grains with a protein content of 10.7%. Similarly, the second set of germplasm consisted of the *GpcB1* gene and *Yr15* in the PBW550 background (PBW550 + *GpcB1+Yr36+ Yr15*) in the F_6_ generation. Out of 81 available lines having the same genetic composition, the third line (F_6_ 3) was used for crossing as parent 2 owing to its better agronomic performance, high protein content, and low polyphenol oxidase activity (Supplementary Table S2, Gill M S, 2017). PBW550 (WH594/RAJ3856//W485) is a double dwarf early maturing bread wheat variety developed and released by Punjab Agricultural University, Ludhiana (notification number 72 (E) dated 10.01.2008, Indian Council of Agricultural Research (ICAR), Government of India) for timely sown irrigated conditions in the northwestern plain zone of India having good grain and *chapati* quality.

### 2.2 Combining high carotenoid, high grain protein content, and rust resistance

A stepwise hybridization and phenotypic-biochemical-molecular selection strategy in the introgression program were followed to obtain agronomically elite plants with desirable genes under consideration. The germplasm lines of parent 1 and parent 2 were validated using biochemical and molecular analysis for carotenoid content, protein content, and rust resistance genes. Eight individual crosses were made between parent 1 and parent 2 where eight different lines with the same genetic composition (HD2967 + *PsyE1/Sr25/Lr19*) were used as parent 1 against single parent 2 (‘F_6_ 3’ having genetic composition PBW550 + *GpcB1+Yr36+ Yr15*), during the main season (November to May) at ‘Wheat Experimental Area’, Punjab Agricultural University, Ludhiana (30.9010° N, 75.8573° E, 810 ft above msl) in 2017–18 (Table 1). F_1’_s were raised at the off-season (June to October) location of Punjab Agricultural University, Ludhiana situated in the Himalayas at Keylong, Lahaul&Spiti District, Himachal Pradesh (32.210° N, 77.140° E, 10,500 ft above msl). Off-season or alternate season refers to production outside their typical cropping cycle and is used to advance the generations mainly by the breeders.

**TABLE 1:** Crosses made for pyramiding *PsyE1* gene and *GpcB1* gene.

Pyramiding *PsyE1* gene, *GpcB1* and rust resistance	
1	BC_1_F_5_ 14X F_6_ 3 (F_1_ Died)
2	BC_1_F_5_ 18X F_6_ 3
3	BC_1_F_5_ 20X F_6_ 3
4	BC_1_F_5_ 23X F_6_ 3
5	BC_1_F_5_ 70X F_6_ 3 (F_1_ Died)
6	BC_1_F_5_ 99X F_6_ 3 (F_1_ Died)
7	BC_1_F_5_ 124X F_6_ 3 (F_1_ Died)
8	BC_1_F_5_ 154X F_6_ 3

F_2_ was raised at Ludhiana during the main crop season in 2018–19. The selection of phenotypically promising rust-resistant single plants was done in artificially inoculated conditions for stripe rust and leaf rust. Grains from selected plants were screened for carotenoid pigments using spectrophotometric evaluation and high grain protein content (GPC) using the Kjeldahl method as well as for the genes of interest using MAS. Selected single plant progenies along with parental lines HD2967 and PBW550 were planted in 1.5 m paired rows with a plant-to-plant distance of 10 cm and row-to-row distance of 20 cm in a randomized block design. The selected lines were carried forward to F_3_/F_4_/F_5_ in 2019–20, 2020–21, and 2021–22, respectively. In each generation, plant/progenies selection was based on rust resistance, carotenoid content, and GPC. Four promising lines having all the desired genes combined with elite agronomic traits and grain quality (luster, boldness, thousand-grain weight, etc.) were identified for evaluation of yield potential.

### 2.3 Screening against foliar rust resistance

Screening for stripe rust and leaf rust was performed in the field along with MAS to validate the effectiveness of rust resistance genes linked to gene *GpcB1*(*Yr36*) and *PsyE1 (Lr19)* as well as for *Yr15*. Progenies from F_2_/F_3_/F_4_ and F_5_ generations were screened against stripe rust and leaf rust at the adult plant stage in the field during seasons 2017–18 to 2021–22. For screening, artificial epiphytotic conditions for rust were created by spraying the urediniospores of *Pt* pathotypes (100S119, 78S84), and *Pt* pathotypes (77–1, 77–2, 77–5, 104–2) diluted in water containing Tween-20. For the uniform spread of disease, highly susceptible cultivars HD2967, PBW343, and PBW550 were planted as spreader rows all around the field and after every 20 rows (Kaur et al., 2020). Data was recorded using the Cobb’s scale, as illustrated by Peterson et al., (1948), when ‘check’ cultivars showed complete susceptibility. The screening for stem rust could not be done in field conditions in Punjab since it is not prevalent in the region and hence, it was monitored using linked molecular markers only. As *Sr25* is linked to *PsyE1*, therefore, the positive selection for *PsyE1* implies the presence of *Sr25*.

### 2.4 Biochemical analyses

#### 2.4.1 Protein content estimation

The Kjeldahl method was used for protein content estimation. 100–200 mg of seeds were weighed. Digestion mix (K_2_SO_4_:Cu_2_SO_4_ in a 9:1 ratio) and concentrated H_2_SO_4_ were added to the samples and placed in the digestion tubes. The tubes were then placed in the digestion assembly for 30 min at 420°C. 25 mL of distilled water was added to the mixture after cooling it to room temperature. In a flask, 0.01N HCl was added followed by three drops of acetocarmine. Distillation was then performed for 3 min by placing the sample tubes with 20 mL of added alkali and the flask in the distillation chamber. The flask was then titrated with the help of a 0.1N NaOH solution. The amount of solution required to change the color of the solution of the flask from pink to yellow was recorded. The protein content was calculated by the formula:

Protein content= (0.08092 x volume of NaOH used for titration)/weight of the sample (Gill, 2017).

#### 2.4.2 Extraction and estimation of total carotenoid

A grounded wheat grain sample (4 g) was taken in a glass test tube, followed by the addition of 20 mL water-saturated butanol. The sample was mixed and shaken properly. The tube was then covered with aluminum foil and kept overnight in the dark at room temperature. All the samples were filtered using Whatman filter paper No. 1 and were allowed to stand for 20 min at room temperature. Absorbance was recorded in a spectrophotometer at 440 nm and contents were expressed in ppm per gram of dry weight as per the following formula:

Carotenoid content (ppm) = [(O.D. X 23.5366) + 0.0105] (Mishra and Gupta, 1995).

#### 2.4.3 Phenol test

Nearly 30 seeds were placed in the Petri plates containing water in the evening and incubated overnight. Excess water was decanted the next morning, followed by the addition of 1% phenol solution (prepared by mixing Phenol crystalline AR grade, molecular weight 94.11 g solvent into double distilled water) and kept for 4 h. The solution was decanted and the seeds were then scored from 1 to 10 based on the extent of coloring within 1h of decanting (Kaur et al., 2013).

### 2.5 DNA extraction and marker-assisted selection (MAS) for *PsyE1/Lr19/Sr25, GpC-B1/Yr36,* and *Yr15*

The selection for the traits i.e., high carotenoid content, high protein content, and resistance to foliar rusts was achieved in a combined phenotypic—genotypic strategy. For the marker-assisted selection, the DNA of F_2_ plants was extracted from young leaves using the CTAB method (Saghai-Maroof et al., 1984) with some modifications (Kaur et al., 2020). *PsyE1/Lr19/Sr25* was screened using a combination of gene-specific markers i.e., Psy1-E1 specific and Psy1-D1 specific (Zhang and Dubcovsky 2008). F_2_s were screened for *GpC-B1/Yr36* using the dominant marker Xucw108 (Uauy et al., 2006). Similarly, the rust-resistant gene, *Yr15,* was screened with marker Xbarc8 (https://wheat.pw.usda.gov/) using specific primers (Table 2). The primers were used to amplify the desired DNA segment using specific PCR (polymerase chain reaction) profiles (Supplementary Table S1) and separated by gel electrophoresis using 2% agarose gel in 0.5X TBE buffer. The amplicons (Table 2) were visualized in a Biorad-gel-documentation system with an ethidium bromide stain.

**TABLE 2:** Markers, their sequences used for Marker Assisted Selection.

S. No.	Genes (markers)	Chromosome location	Primer sequence	Marker type	Amplicon size	References
1	*Yr15*	1BS	*Xbarc8*	Dominant	245 bp	https://wheat.pw.usda.gov/
			*5′GCG​GGA​ATC​ATG​CAT​AGG​AAA​ACA​GAA 3′*			
			*5′GCG​GGG​GCG​AAA​CAT​ACA​CAT​AAA​AAC​A 3′*			
2	*Yr36/GpC-B1*	6BS	*Xucw 108*	Dominant	217 bp	Uauy et al (2006)
			5′ ATC​TGC​AAT​TCC​AGG​CAC​AC 3′			
			5′ CCA​GCA​GAT​CAA​GGA​GAA​TTG 3′			
3	*PSY1-D1 Specific*	7DL	5′*TTG​CAG​TGC​AAT​GGT​TTT​CCA*3′	Dominant	∼175 bp	Zhang and Dubcovsky (2008)
			5′*GAC​TCC​TTT​GAC​GAT​GTC​TTC*3′			
4	*PSY1-E1 Specific*	7AL/7DL/7 EL	5′*CTA​CGT​TGC​GGG​CAC​CGT​T*3′	Dominant	∼191 bp	Zhang and Dubcovsky (2008)
			5′*AGA​GAA​AAC​CAT​TGC​ATC​TGT​A*3′			

## 3 Results

### 3.1 Development of segregating population for gene pyramiding

**FIGURE 1:**
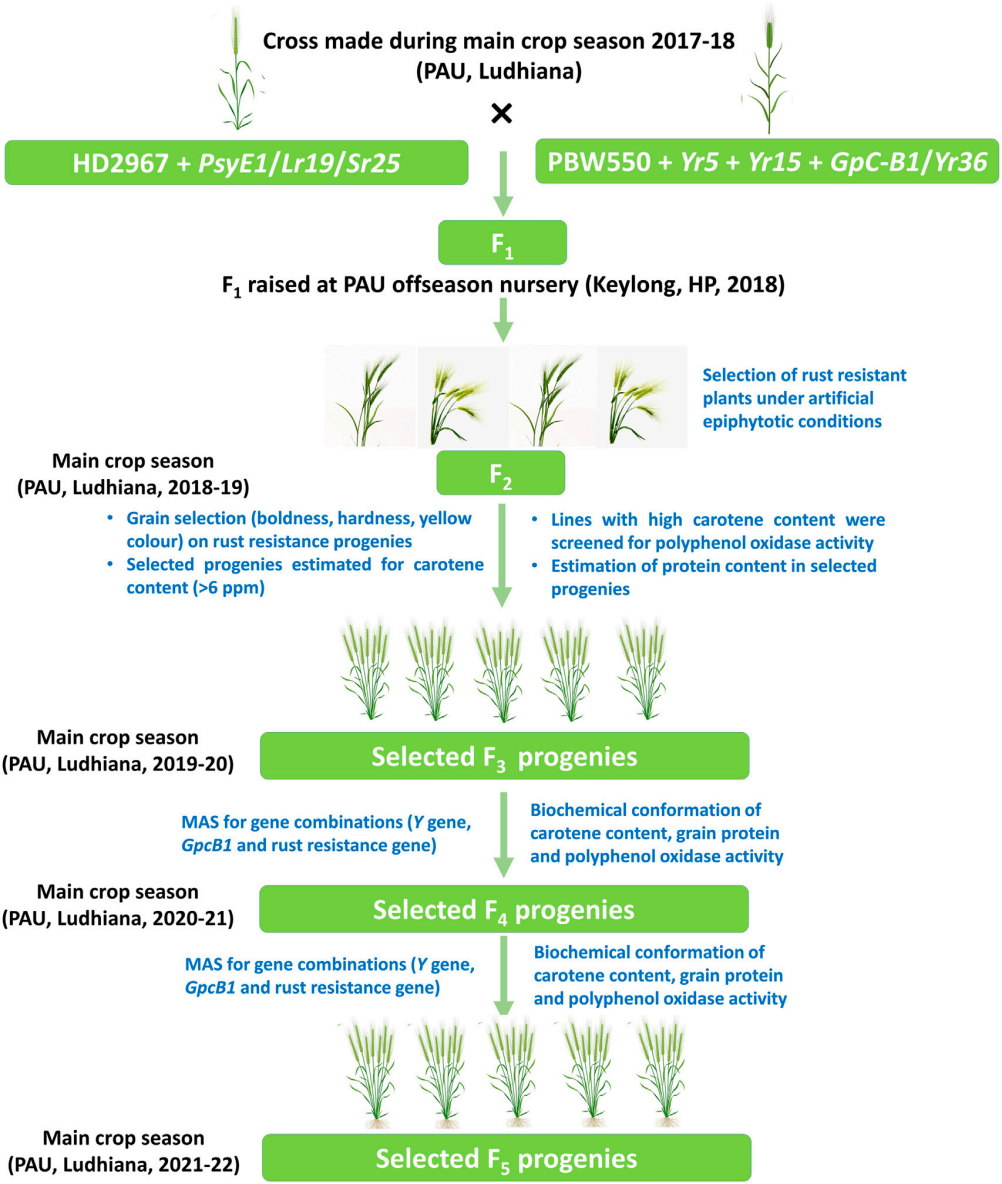
Representation of the steps performed for pyramiding PBW550 + *GpcB1/Yr36 + Yr15*and HD2967 + *PsyE1/Lr19/Sr25*.

Based on the screening of individual populations for high protein content (PBW 550 + *Yr15 + GpcB1/Yr36*) and high carotenoid content (HD 2967 + *PsyE1/Sr25/Lr19*), the phenotypic expression of both *PsyE1* and *GpcB1*was observed to be variable among the plants, as guided by their genotype constitution. Therefore, it is necessary to choose the line with the best agronomic characters as the parental type for developing the segregating population. Amongst the eight crosses made among selected lines, four crosses (i.e. BC_1_F_5_ 20/F_6_ 3, BC_1_F_5_ 18/F_6_ 3, BC_1_F_5_ 154/F_6_ 3, BC_1_F_5_ 23/F_6_ 3) survived, while the F_1_generation of the remaining crosses suffered hybrid necrosis due to low temperature and the genetic makeup of the cultivar HD2967 (Supplementary Figure S1) (Mishra et al., 2017; Takumi and Mizuno, 2011). Hybrid necrosis (gradual premature death of leaves or plants in certain wheat F_1_ hybrids) is caused by the interaction of two dominant complementary genes, *Ne1* and *Ne2,* located on chromosome arms 5BL and 2BS, respectively (Chu et al., 2006) and the HD2967 genotype carrying both the genes is often reported to cause hybrid necrosis in F_1_s. The seeds harvested from the remaining four crosses were sown as a single seed to raise a total of 2748 F_2_ individuals which were phenotyped for rust resistance (stripe rust and leaf rust) along with yellow grain pigment content. A subset of 649 F_2_ plants was selected for further screening of biochemical constituents (carotenoid content, protein content, and polyphenol oxidase activity) and MAS (using gene-specific markers for the genes, *Yr15, GpcB1/Yr36* and *PsyE1/Sr25/Lr19*) (Figure 1).

### 3.2 Marker-assisted selection (MAS)

MAS was carried out to select the genotypes combining *PsyE1/Sr25/Lr19*, *GpcB1/Yr36,* and two stripe-rust-resistant genes along with *Yr15* in the F_2_ plants sown as single plants. The four crosses which survived made up a total of 2748 F_2_ individual plants. The conventional MAS was modified to achieve the best results out of the selected F_2_ plants. For the gene pyramiding experiments, expression of the genes in terms of the phenotype is equally important as their presence in a genotype. Therefore, sequential screening was performed for rust resistance (stripe rust and leaf rust) followed by estimation of carotenoid content and protein content before molecular validation to minimize the time, effort, and cost for the selection of pyramided plants (Figure 1).

#### 3.2.1 Screening for rust resistance

All the F_2_ individual plants were screened for stripe rust and leaf rust resistance under artificial epidemic conditions as described in Section 2.3 (material and methods). All the individual plants with an infection of any kind of rust or both were uprooted and discarded before proceeding to the selection for carotenoid content. The leaf tissue was collected from the remaining 1735 individual plants out of the total of 2748 F_2_s after screening for rust resistance.

#### 3.2.2 Screening for carotenoid content

**FIGURE 2:**
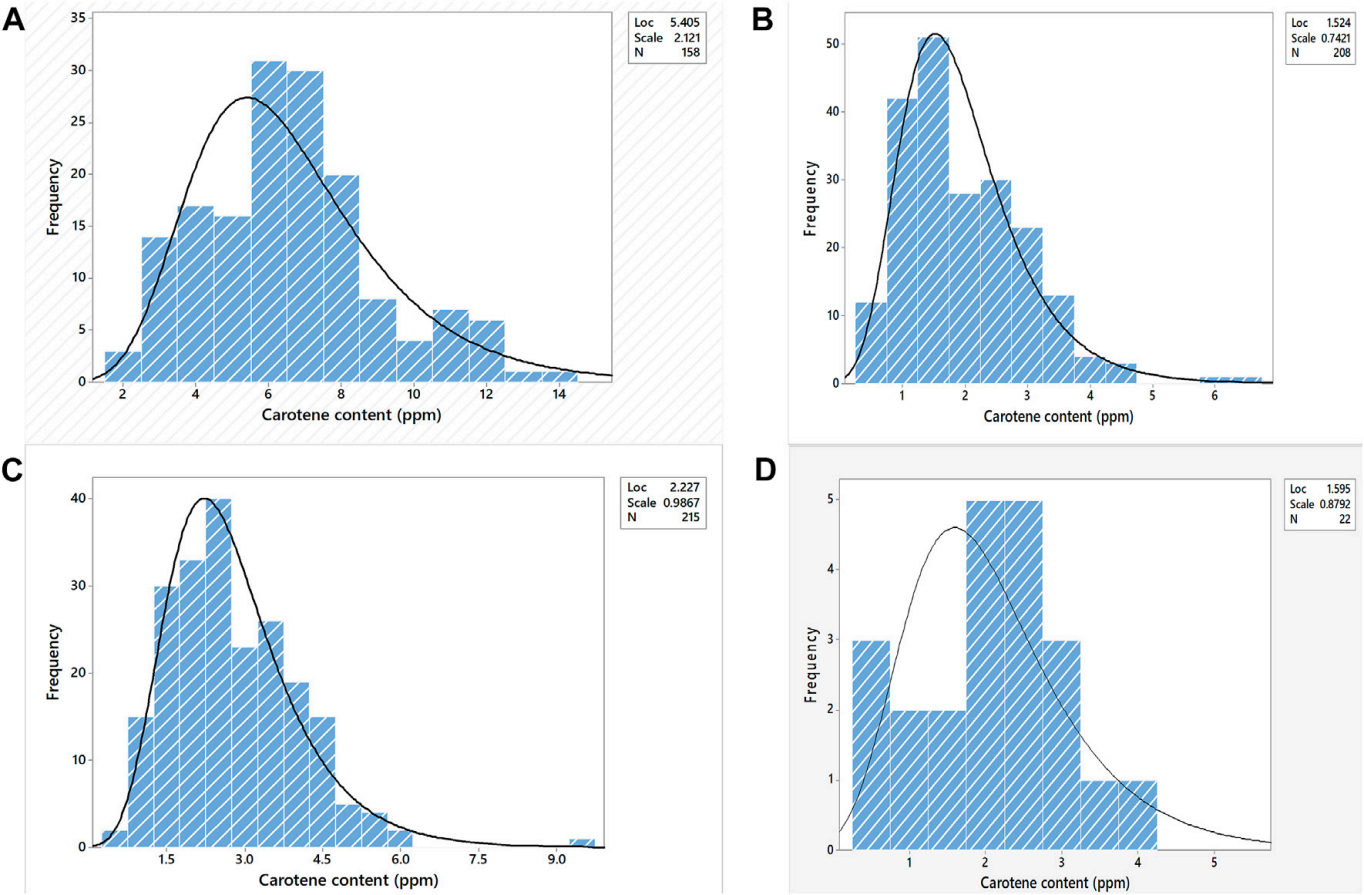
Frequency distribution of carotenoid content in the F2 population of the cross **(A)**. BC_1_F_5_ 154X F_6_ 3, **(B)**. BC_1_F_5_ 23X F_6_ 3, **(C)**. BC_1_F_5_ 20X F_6_ 3, **(D)**. BC_1_F_5_ 18 X F_6_ 3 where “Loc” (location) specifies the Mean, “Scale” specifies the Standard Deviation, and “N” specifies the number of individuals taken under consideration.

The *Y* gene (*PsyE1*) derived from *Thinopyrum ponticum* encodes for phytoene synthase which increases the grain carotenoid content. As we adopted the product-oriented methodology i.e., a combined phenotypic - genotypic strategy in the pyramiding experiment as described previously, we aimed for the expression of the *PsyE1* gene i.e., yellow coloration of the grains before proceeding with the molecular selection based on the *PsyE1* gene. The selection was made for bold, hard, and lustered grains with bright to dark yellow color from the rust-resistant plants followed by biochemical selection for carotenoid content in the grains. For the cross BC_1_F_5_ 20/F_6_ 3, having 732 F_2_ plants, a total of 232 plants were selected on the field in the manual selection (seed color giving the preliminary indication) followed by biochemical estimation and being observed to have carotenoid content in the range of 0.59–9.49 ppm (Supplementary Table S6). For the cross BC_1_F_5_ 18/F_6_ 3, among the selected 22 plants out of 458 F_2_ plants, carotenoids were estimated in the range of 0.47–4.14 ppm (Supplementary Table S3). Similarly, 177 F_2_ plants in the cross BC_1_F_5_ 154/F_6_ 3, were selected with 1.58–12.17 ppm carotenoid content, from a total of 624 plants (Supplementary Table S4).In the cross, BC_1_F_5_ 23/F_6_ 3, out of 934 plants, 218 F_2_ plants having grain carotenoid in the range of 0.45–6.55 ppm were selected (Supplementary Table S5). The grains with >6 ppm (almost double the carotenoid content of the available commercial bread wheat varieties) of carotenoid content were selected, to further reduce the number of selected F_2_s for molecular screening (Figure 2). In this process only, BC_1_F_5_ 154/F_6_ 3 cross was selected as it yielded seeds possessing a gradient of carotenoid content in the range of 1.58–12.17 ppm. In the remaining crosses, the carotenoid content was too small (<6.00 ppm). Out of the selected 177 single plants from the cross BC_1_F_5_ 154/F_6_ 3, 89 plants were chosen after considering the carotenoid content data obtained from them (Supplementary Table S8).

#### 3.2.3 Biochemical screening

Polyphenol oxidases oxidize phenols to cause enzymatic browning and forming dark pigments in unfermented stored flours and dough causing a major perceived aesthetic quality loss in bread wheat (https://maswheat.ucdavis.edu/protocols/PPO). It constitutes a major proportion of wheat proteins besides gluten. Qualitative estimation of polyphenol oxidase activity conducted using a phenol test on 89 selected plants revealed scores in the range of 1.4–4.8 (Supplementary Table S8) and was followed by the selection of 15 plants with scores <3.0, i.e., low polyphenol oxidase activity (Supplementary Figure S1, Supplementary Table S9). To evaluate *GpcB1* gene expression, the protein content was estimated in the above-mentioned 15 selected plants by the Kjeldahl method which was recorded in the range of 11.52%–14.53% (Supplementary Table S9). The plants with >13% of grain protein were advanced for further evaluation and yield trials.

#### 3.2.4 Molecular marker-assisted validation for gene pyramiding

**FIGURE 3:**
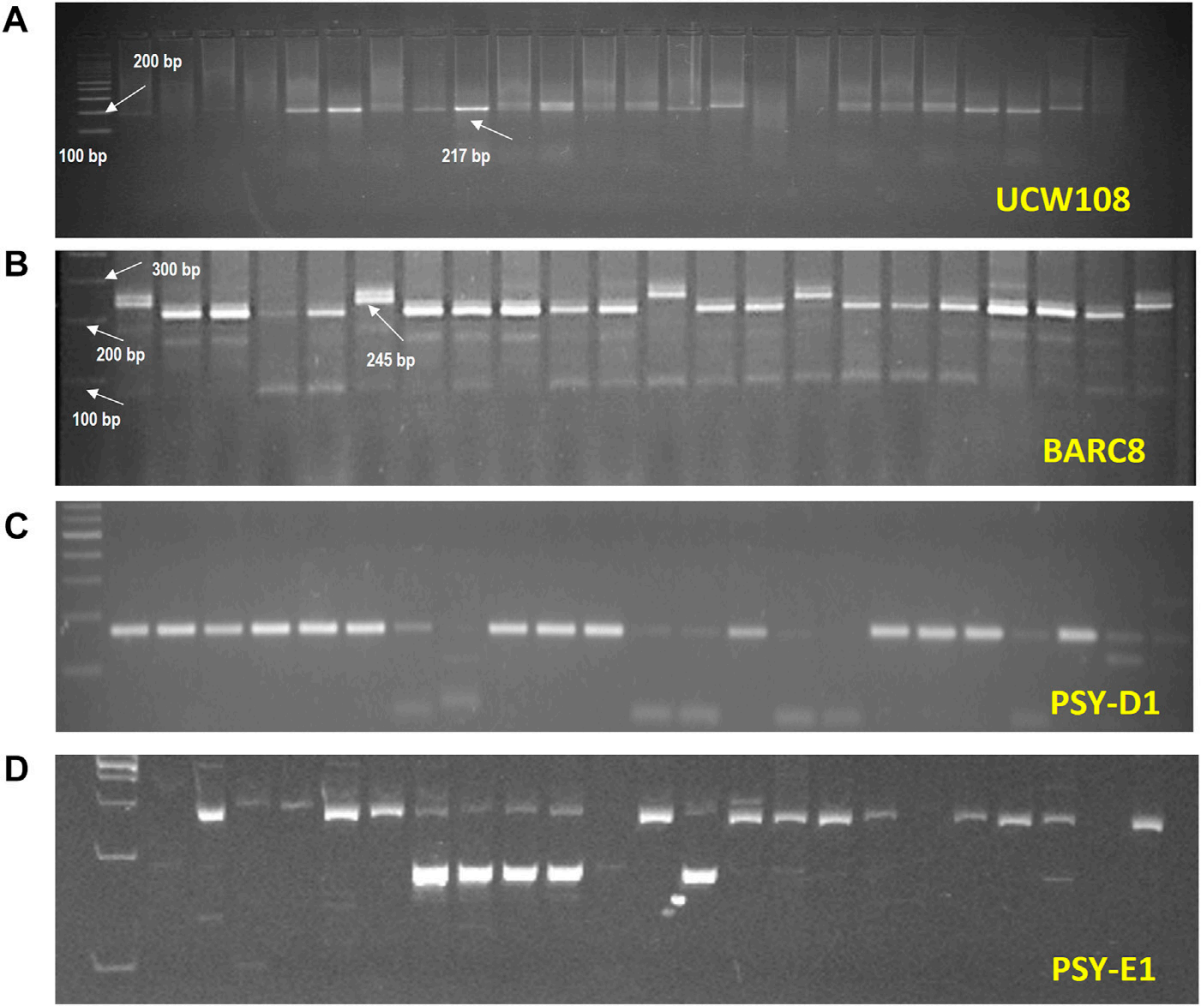
Amplification of markers, **(A)**. UCW108, **(B)**. BARC8, **(C)**. PsyD1 and **(D)**. PsyE1 in the experiments.

The molecular marker “PsyE1 specific” (for *Y* gene) was used to validate the presence of the *PsyE1* gene in all the selected 89 plants, which also screened positive for carotenoid content and gave a band of 191bp (Figure 3). It was observed that all the selected 89 genotypes were positive for the *PsyE1* gene and, hence, were further subjected to screening for Xbarc8, a marker for the rust-resistant gene *Yr15*. As the F_2_ plants were initially screened for rust resistance, it was reflected in the molecular analysis, providing an increased probability of getting positive results for the presence of the rust resistance genes. Xbarc8 produced a band of 245bp in a total of 82 plants, demonstrating these lines carried the *Yr15* gene. As mentioned above, all the selected F_2_s were positive for the *PsyE1* gene and were therefore counted as positive for stem rust and leaf rust resistance genes, i.e. *Sr25* and *Lr19,* respectively, due to their strong linkage with *PsyE1*. Screening with the molecular marker Xucw108 was carried out for validating the presence of the *GpcB1* gene and produced a 217bp band in all the positive lines (Figure 3). Molecular analysis identified 4 plants i.e., plant no. 10, 31, 104, and 160 (Supplementary Table S9) to be positive for all the desired genes. These lines were advanced in the ear-to-row method and the same selection protocol was followed i.e., morphological, biochemical, and molecular selection until the F_5_ generation.

## 4 Discussion

Food and nutritional security, especially in developing countries have gained much significance in the recent past and all major breeding programs have diverted their efforts to not only increase food production but also to improve the nutritional value of the food products simultaneously. India has been producing sufficient wheat to contribute towards food security in the recent past but is still unable to cater to the undernourishment and malnutrition issues, especially for the masses. However, a decline in the undernourished population was witnessed, although a reverse trend started again after 2015, so that currently 9%–11% of the world’s population suffers from undernourishment/malnutrition, and this is likely to increase further (by∼0.8%–1.0%) by 2030 when >850 million people are predicted to suffer with hunger and undernourishment/malnutrition (Purugganan and Jackson 2021; Gupta et al., 2022). The wheat crop deserves major attention as it is the second most important crop after maize in terms of staple food and the second most important crop after rice in terms of food and nutritional security. Many efforts are underway to combine different subcomponents of nutritional quality in wheat, particularly in terms of grain protein content (GPC), iron content, zinc content, and antioxidant content (Gupta et al., 2020; Gupta et al., 2021; Gupta et al., 2022). A novel component of biofortification in wheat other than enhancing iron or zinc content is the enhancement of carotenoid pigments in the mature grain. Since cereals are characterized by low carotenoid content, their biofortification to meet daily human requirements offers an opportunity to combat wide-scale malnutrition deficiencies without changing the diet structures of the population. Besides improved nutritional quality, the yield potential and the disease resistance to major prevalent diseases are also important for developing suitable end-product-specific wheat cultivars. Therefore, the present study focuses on combining high carotenoid content, grain protein content, and disease resistance in high-yielding backgrounds using breeding, biochemical, and molecular techniques.

Marker-assisted selection (MAS) is routinely used to supplement conventional plant breeding (Gupta et al., 2022). MAS-assisted gene pyramiding allows the stacking of desirable genes/alleles of multiple genes for one or more traits in an elite genotype of interest. This method is well demonstrated in numerous crop species to further improve an existing elite cultivar through the introgression of multiple genes from one or more donors. Theoretically, it can be achieved by a single cross provided that the segregating population is large enough (Xu, 2013). As a large population is not always practically feasible, it can be done in two steps by selecting desirable heterozygous/homozygous alleles followed by their self or back-crossing (Ishii and Yonezawa, 2007). In wheat, several successful examples of gene pyramiding are available in a number of varieties (Gupta et al., 2010). With an increasing population and *per capita* income, demand for quality wheat and its products is increasing especially in developing countries. Although conventional breeding approaches have been utilized in developing cultivars with improved quality traits, they are limited by their time demands. With the advent of molecular tools, high throughput and cost-effective markers have been designed to aid in the crop improvement process and have yielded numerous successful examples in wheat itself (Gautam et al., 2020; Liu et al., 2020; Sharma et al., 2021; Awan et al., 2017).

### 4.1 Carotenoid and protein content in biofortified wheat

*PsyE1* gene introgression resulted in a significant increase in the carotenoid content, which varied up to 9.49 ppm in the selected pyramided lines (Supplementary Table S6). Further, the *GpcB1* gene was pyramided with *PsyE1*in the current experiment*,* and the former was observed to increase the protein content up to 14% and 13.5% with and without yield penalty, respectively. The gene also resulted in increased remobilization of nutrients, thereby increasing the Fe and Zn concentration by 10% and 5%, respectively (Uauy et al., 2006). Uauy et al., 2006 first cloned and characterized the grain protein content associated with gene *GpcB1* as well as developed the functional markers which have opened numerous avenues to further increase the nutritional significance of bread wheat. The study also showed that *GpcB1* is also responsible for increased iron and zinc content in grain. Although the expression of this gene is influenced by the environment with a coupled yield penalty (Brevis and Dubcovsky, 2010), examples of introgression of the *GpcB1* gene through MAS without compromising crop yield are also available (Uauy et al., 2006; Brevis and Dubcovsky, 2010). However, *GpcB1*-induced accelerated senescence resulting in shriveled grain seems to be the significant reason that even after widespread use of this gene in almost all wheat breeding programs for a decade, no commercially released variety has this gene introgressed in it. In the present study, the GPC was increased by 2% of existing content, making a total of 13%–14% with minimal yield compromise along with the carotenoid content raised up to 8ppm.

### 4.2 Consumer acceptance of carotenoid-fortified wheat

The yellow color in wheat is controlled by the Phytoene Synthase (*Psy*) gene and its alleles which produces the enzyme. Phytoene Synthase dimerizes two geranylgeranyl pyrophosphate molecules and is also the rate-limiting factor in the carotenoid biosynthesis pathway (Lindgren, 2003). The wheat *Psy1* gene shows homology with the maize *Psy1* gene and is associated with carotenoid accumulation (Palaisa et al., 2003) in the endosperm. In maize, it was mapped on chromosome 6 which shows synteny with chromosome 7 of bread wheat (Gale and Devos, 1998; Palaisa et al., 2003). In wheat, the gene is present in all three homologs of chromosome 7 (*PsyA1, PsyD1,* and *PsyB1*) (Zhang and Dubcovsky, 2008). The original 7E chromosomal segment from *Th. ponticum* was incorporated into the wheat cultivar “Agatha” containing the *Psy* gene which was translocated into chromosomes 7A and 7D of wheat and was named the *Y* gene due to the undesired yellow coloration of the flour (Sharma and Knott, 1966). Plants heterozygous for the 7 EL segment showed 56% higher lutein content than the lines without the 7 EL segment (Zhang et al., 2005). Carotenoid (lutein) is the main component mediating the yellow coloration of wheat flour (Adom et al., 2003; Zhang et al., 2005). Further, the yellow color of the flour is desirable for yellow alkaline noodles consumed mostly in Japan and Southeast Asia (Kruger et al., 1992). Similarly, in North India, *missi roti* is consumed, by adding chickpea flour to wheat flour for added taste and colour is quite popular (Padhy et al., 2022b; Padhy, 2019).

### 4.3 Rust resistance in biofortified wheat

In our experiment, the genes *PsyE1+Lr19+Sr25* in HD2967 background with *GpcB1+Yr36+ Yr15* in PBW550 background were converged and stacked together. After the selection of the desirable lines utilizing gene-specific markers, the resultant lines showed total rust resistance to both stripe and leaf rust, thus demonstrating the expression and functionality of the *Lr19, Yr36,* and *Yr15* genes. *Yr36* is the temperature-sensitive stripe rust resistance gene which confers enhanced resistance to stripe rust as the temperature increases. Combining one major stripe rust resistance gene (*Yr15*) which ensures completely clean plant foliage with temperature sensitive *Yr36* gene takes care of stripe rust resistance. Further, *Lr19,* linked to the *Y* gene, partially covers leaf rust. The other minor/defeated leaf rust genes in several backgrounds may add to the complete resistance when combined with other genes. This kind of additive effect of minor defeated genes has been also shown to provide resistance in segregating generations of two susceptible cultivars who reported that in the two crosses (PBW621×PBW343 and HD2967×PBW343) the resistant segregants possessed two genes, one contributed by PBW621 or HD2967 (depending on the cross) and the other, unexpectedly but obviously, came from the most susceptible cultivar, PBW 343 (Sharma et al., 2022). Also, the gene *Sr25,* linked to the *Y* gene, makes a case for pre-emptive breeding for black rust in wheat.

### 4.4 Yield and agronomic traits of carotenoid biofortified wheat

An increased carotenoid content (up to 12ppm) in the segregating population was limited by a yield penalty, such as reducing the number of tillers in the lines than the checks. However, it was observed that the lines pyramiding all the desired genes had carotenoid content up to 9 ppm without any substantial yield penalty. An increased carotenoid content of 50%–120% was observed in these pyramided lines as compared to those previously reported by Zhang et al. (2005), which showed 56% higher lutein content than the lines without the 7 EL segment. The agronomic characterization of the translocated isogenic lines demonstrated that the presence of 7E was associated with high grain yield under irrigated conditions (Monneveux et al., 2003). After the characterization of the gene by Zhang and Dubcovsky, (2008), the gene was used positively for the first time in a durum background by Singh et al. (2014). Sun et al. (2014) found a significant negative correlation between carotenoid content and thousand-grain weight and a positive correlation between carotenoid content and grain length, grain length/grain width, and grain length/grain thickness. They also reported a negative correlation between carotenoid content and grain width and grain thickness. Similarly, Padhy A. K. et al. (2022) reported a significant negative correlation between carotenoid content and thousand-grain weight and spike length. Spikelets per spike and grains per spike were negatively correlated with carotenoid content but were insignificant. However, they reported a slight positive correlation between carotenoid content and tillers per meter row as well as yield. It implies that the *Y* gene can be effectively used for inbreeding wheat with high carotenoid content without any major yield penalty provided the population size is kept large so as not to lose out the rare recombinants combining high carotenoid content, high grain weight, and disease resistance.

### 4.5 Health benefits of biofortified wheat

Micronutrient deficiencies affect a large section of the world’s population. Biofortification is an evidence-based nutrition strategy to address some of the most common and preventable global micronutrient gaps and can help improve consumer health (Bouis and Saltzman, 2017; Foley et al., 2021). Enhancing the micronutrient concentration, specifically protein, iron, and zinc content in wheat *via* genetic fortification using modern plant breeding approaches has been exploited as the most effective and safe approach to alleviate micronutrient malnutrition by plant breeders. Further, a number of issues concerning the nutritional quality of wheat revolve around the seed phosphorus (P) storage compound called phytic acid (myo-inositol-1,2,3,4,5,6-hexakisphosphate) for which studies report genetic variability in available wheat germplasm (Sharma et al., 2022). Enhancing carotenoid content in wheat is a novel addition to biofortification components to be considered when breeding biofortified wheat cultivars (Padhy et al., 2022b). Lutein, the major component of carotenoids, is an antioxidant that reduces oxidative damage to biological membranes by scavenging peroxy-radicals, which are involved in certain human diseases and aging, as well as responsible for the degeneration of food quality. Therefore, the carotenoid pigments contribute to an increased nutritional value of wheat and wheat products (Bast et al., 1996). The *PsyE1* gene is present in the distal region of the chromosome arm 7 EL from *Th. ponticum* increases carotenoids in the endosperm and is linked with the rust-resistant genes *Lr19* and *Sr25* (Zhang et al., 2005; Padhy et al., 2022a). The incorporation of the *PsyE1* gene increases all the carotenoids that are produced in the carotenoid biosynthesis pathway. There is an increase in lutein, zeaxanthin, and β-carotein content due to the introgression of *PsyE1* (Zhang et al., 2005; Ceoloni et al., 2017; Padhy A. K. et al., 2022). In a bread wheat background, despite the numerous health benefits of carotenoids, the gene was never used commercially as the yellow coloration of wheat flour lacked consumer acceptability and health-associated awareness. But recent trends and demand for biofortified quality wheat and wheat products have encouraged us to use the gene positively by analyzing its role in increased carotenoid content, agronomic effects, and potential health benefits (Padhy et al., 2022b).

Wheat breeding in combination with advances in biotechnology tools has made remarkable progress in increasing crop yields in the recent past. Wheat breeders must constantly respond to ever-emerging challenges, primarily, nutritional security, which defines the major mandate of a wheat breeder in the present scenario. Wheat breeders in India have always faced the endless task of continually developing new wheat varieties combining enhanced yield and disease resistance and have emerged successful, leading to self-sufficiency in wheat. However, in light of the malnourishment statistics of the country, the task of combining two or more nutritional components into agronomically elite backgrounds, and, if not enhancing, at least maintaining substantial yields represents an unprecedented challenge for wheat breeders. Varieties with improved nutritional quality, protein content, high grain yield, high carotenoid content, and desirable processing quality in adapted elite genetic backgrounds with tolerance to stresses and diseases can help alleviate nutrient deficiencies. Breeding wheat with enhanced levels of nutritional components is a cost-effective, sustainable solution to malnutrition problems. It is therefore paramount that suitable biofortified wheat varieties are developed, released, and disseminated for widespread adoption. Since grain nutrition is a non-visible trait, it is essential that new cultivars not only have enhanced nutritional components but also higher yield levels.

### 4.6 Shuttle breeding for combining quality components in wheat

Shuttle breeding, started at CIMMYT, Mexico, was originally used to accelerate the breeding cycle by growing segregating generations in contrasting environments. This could ensure rapid generation advance by taking more than a single generation per year. Additional benefits of shuttle breeding were observed in terms of wider as well stringent selection due to exposure of the breeding material to variable disease spectrums, soil types, photoperiod lengths, and diverse abiotic stresses. Wheat breeders in North India, including Punjab Agricultural University located in India, Punjab, exploit the shuttle breeding concept and grow alternate wheat crops at Keylong, situated in the Himalayas. This has been highly effective in achieving the breeding objectives as the wheat after harvesting (end of April to first fortnight of May) from the main crop season is planted at an alternate location or commonly known as the off-season location at Keylong in the second fortnight of May. The crop is harvested from Keylong at the end of September and the seed is ready to be sown back in the main crop season in November. This cycle fits well for major breeding objectives focusing on grain yield. However, when the breeding is aimed at stacking together the various subcomponents of quality, this system poses a major challenge in terms of the time available for quality component analysis in the screening of the generations before the next sowings. Wheat grain quality primarily refers to two main components, namely the nutritional content (which may be for domestic or commercial use) and industrial processing or end product specificity. Enhancing wheat quality improves processing specificity, makes more desirable and more diverse consumer products and ensures the competitiveness of farmers, grain merchandisers, millers, and end processors. Wheat quality criteria may vary drastically depending on the end use. Usually, the conventional small-scale quality tests and/or marker-assisted selection (MAS) are done in the advanced breeding stages (F_6_–F_8_) or the final product. But combining together different components of quality definitely requires stringent screening for the traits under consideration from the F_2_ generation onwards. Moreover, this testing must be based on more specific food processing (dough visco-elasticity and mixing properties, starch pasting properties, baking performance, etc.). Since the breeding for complex multigenic quality characters in wheat by biochemical analyses and functional pilot tests is traditionally a slow process, shuttle breeding has limited utility. Instead, rapid generation advance (RGA) under controlled conditions or doubled haploid production can be of much use. This explains the breeding scheme presented here which uses an off-season location for advancing F_1_ to F_2_ only and the other generations were evaluated for selection in the main season only.

## 5 Conclusion

In conclusion, the present study is a successful example of seamlessly combining conventional breeding and biotechnological tools (MAS) for pyramiding diverse subcomponents of grain fortification and disease resistance in wheat. The exploitation of the *Y* gene in a constructive way to increase grain pigment content is in contrast to the earlier approaches (Sharma and Knott, 1966; Sears, 1972) that focused on dissecting the gene by only retaining the linked rust-resistant genes. This exploitation was coupled with enhancing grain protein as well as micronutrient content in an innovative attempt at wheat breeding. The parental material with different components of quality, fortification, or resistance individually was available in the background of the popular wheat varieties (HD2967 and PBW550), which hold the honor of being the mega-varieties adapted to various wheat growing agro-climatic zones of India, such that their contribution to national food security is significant. In the present study, the development of advanced wheat lines with high carotenoid content in the grains, increased protein content, rust resistance, and elite plant type is reported. These lines will be very useful as new genetic resources for future wheat breeding programs. The improved lines will be further evaluated for their agronomic performance and other yield-related traits in preliminary and multi-location yield trials to examine their potential to provide next–generation improved versions of rust-resistant biofortified wheat with superior grain quality. The derived wheat lines have the potential of developing into newer, biofortified, and improved germplasm for commercial cultivation.

## Data Availability

The original contributions presented in the study are included in the article/Supplementary Material, further inquiries can be directed to the corresponding authors.

## Abbreviations

CIMMYT, *Centro Internacional de Mejoramiento de Maíz y Trigo* or in English, international maize and wheat improvement center, Mexico; FAO, food and agriculture organizatio; ft, foot; g, gram; Gpc, grain protein content; HTAP, high-temperature adult-plant; ICAR, Indian Council of agricultural research, India; Lr, leaf rust; MAS, marker-assisted selection; Msl, mean sea level; O.D, optical density; ppm, parts per million; Psy, phytoene synthase; Pt pathotypes, *Pucciniatriticina* pathotypes; RGA, rapid generation advance; Sr, stem rust; Th, Thinopyrum; *Y* gene, yellow pigment gene (alternatively *PsyE1*); Yr, yellow rust.
