# Supplementary material for: Combining high carotenoid, grain protein content and rust resistance in wheat for food and nutritional security
Source: Front Genet. 2023 Jan 19;14:1075767. doi: 10.3389/fgene.2023.1075767 (PMC9893017; doi:10.3389/fgene.2023.1075767)
Supplement: Supplementary file 1 [file DataSheet2.docx]

Supplementary Tables

**S1: Table of PCR profiles for the markers used in the experiment.**

| **Gene** | **Primer** | **Steps** | **Temperature (°C)** | **Minutes** | **Number of cycles** |
| --- | --- | --- | --- | --- | --- |
| *Yr15* | Xbarc8 | Initial Denaturation | 95 | 6 | 1 |
|  |  | Denaturation | 95 | 1 | 40 |
|  |  | Annealing | 50 | 1 |  |
|  |  | Extension | 72 | 1 |  |
|  |  | Final Extension | 72 | 7 | 1 |
|  |  | Hold | 4 | ∞ |  |
| *Yr36/ GpC- B1* | Xucw 108 | Initial Denaturation | 95 | 6 | 1 |
|  |  | Denaturation | 95 | 1 | 35 |
|  |  | Annealing | 59 | 0:30 |  |
|  |  | Extension | 72 | 1 |  |
|  |  | Final Extension | 72 | 7 | 1 |
|  |  | Hold | 4 | ∞ |  |
| *Psy E1/D1* | Psy E1/D1 | Initial Denaturation | 95 | 6 | 1 |
|  |  | Denaturation | 95 | 0:30 | 10  (Touch Down) |
|  |  | Annealing | 63 | 0:30 |  |
|  |  | Extension | 72 | 1:20 |  |
|  |  | Denaturation | 95 | 0:30 | 35 |
|  |  | Annealing | 58 | 0:30 |  |
|  |  | Extension | 72 | 1:20 |  |
|  |  | Final Extension | 72 | 7 | 1 |
|  |  | Hold | 15 | ∞ |  |

**S2: Protein content and phenol reaction score of F_6_ (PBW550+*GpcB1*) lines**

| **Sr. No.** | **Parentage** | **Protein (%)** | **Phenol Reaction Score**  **(Out of 10)** |
| --- | --- | --- | --- |
| 1 | PBW550 + *GpcB1/ Yr36 + Yr15* | - | - |
| 2 | PBW550 + *GpcB1/ Yr36 + Yr15* | 13.65 | 2.4 |
| 3 | PBW550 + *GpcB1/ Yr36 + Yr15* | 13.46 | 1.5 |
| 4 | PBW550 + *GpcB1/ Yr36 + Yr15* | 13.80 | 2.5 |
| 5 | PBW550 + *GpcB1/ Yr36 + Yr15* | 13.91 | 2.4 |
| 6 | PBW550 + *GpcB1/ Yr36 + Yr15* | 12.20 | 1.9 |
| 7 | PBW550 + *GpcB1/ Yr36 + Yr15* | 11.57 | 1.5 |
| 8 | PBW550 + *GpcB1/ Yr36 + Yr15* | 11.56 | 2.3 |
| 9 | PBW550 + *GpcB1/ Yr36 + Yr15* | 12.43 | 2.3 |
| 10 | PBW550 + *GpcB1/ Yr36 + Yr15* | 12.32 | 2.1 |
| 11 | PBW550 + *GpcB1/ Yr36 + Yr15* | 12.11 | 2.1 |
| 12 | PBW550 + *GpcB1/ Yr36 + Yr15* | 11.61 | 2.2 |
| 13 | PBW550 + *GpcB1/ Yr36 + Yr15* | 12.36 | 2.2 |
| 14 | PBW550 + *GpcB1/ Yr36 + Yr15* | 13.17 | 2.1 |
| 15 | PBW550 + *GpcB1/ Yr36 + Yr15* | - | - |
| 16 | PBW550 + *GpcB1/ Yr36 + Yr15* | 12.17 | 1.9 |
| 17 | PBW550 + *GpcB1/ Yr36 + Yr15* | 11.87 | 1.7 |
| 18 | PBW550 + *GpcB1/ Yr36 + Yr15* | - | 1.9 |
| 19 | PBW550 + *GpcB1/ Yr36 + Yr15* | 12.40 | 2.5 |
| 20 | PBW550 + *GpcB1/ Yr36 + Yr15* | 12.68 | 3.2 |
| 21 | BWL 5429 (C) | 12.32 | 5.0 |
| 22 | 09014/23(C) | 11.73 | 2.3 |
| 23 | PBW550 + *GpcB1/ Yr36 + Yr15* | 11.97 | 1.9 |
| 24 | PBW550 + *GpcB1/ Yr36 + Yr15* | 12.17 | 2.0 |
| 25 | PBW550 + *GpcB1/ Yr36 + Yr15* | 12.59 | 3.2 |
| 26 | PBW550 + *GpcB1/ Yr36 + Yr15* | 12.79 | 2.2 |
| 27 | PBW550 + *GpcB1/ Yr36 + Yr15* | - | 3.8 |
| 28 | PBW550 + *GpcB1/ Yr36 + Yr15* | - | 2.3 |
| 29 | PBW550 + *GpcB1/ Yr36 + Yr15* | 11.61 | 3.5 |
| 30 | PBW550 + *GpcB1/ Yr36 + Yr15* | 13.52 | 2.1 |
| 31 | PBW550 + *GpcB1/ Yr36 + Yr15* | - | 2.3 |
| 32 | PBW550 + *GpcB1/ Yr36 + Yr15* | 12.44 | 1.9 |
| 33 | PBW550 + *GpcB1/ Yr36 + Yr15* | 13.28 | 2.0 |
| 34 | PBW550 + *GpcB1/ Yr36 + Yr15* | 12.21 | 2.1 |
| 35 | PBW550 + *GpcB1/ Yr36 + Yr15* | 13.94 | 1.9 |
| 36 | PBW550 + *GpcB1/ Yr36 + Yr15* | - | 2.5 |
| 37 | PBW550 + *GpcB1/ Yr36 + Yr15* | 12.45 | 2.3 |
| 38 | PBW550 + *GpcB1/ Yr36 + Yr15* | 12.00 | 2.1 |
| 39 | PBW550 + *GpcB1/ Yr36 + Yr15* | 12.67 | 2.1 |
| 40 | PBW550 + *GpcB1/ Yr36 + Yr15* | 12.70 | 3.4 |
| 41 | PBW550 + *GpcB1/ Yr36 + Yr15* | 13.10 | 3.4 |
| 42 | PBW550 + *GpcB1/ Yr36 + Yr15* | 11.89 | 2.5 |
| 43 | BWL 6209(C) | - | 3.8 |
| 44 | BWL5429 (C) | 12.41 | 5.0 |
| 45 | PBW550 + *GpcB1/ Yr36 + Yr15* | 11.70 | 3.6 |
| 46 | PBW550 + *GpcB1/ Yr36 + Yr15* | 12.93 | 3.4 |
| 47 | PBW550 + *GpcB1/ Yr36 + Yr15* | 12.38 | 2.0 |
| 48 | PBW550 + *GpcB1/ Yr36 + Yr15* | 12.22 | 2.3 |
| 49 | PBW550 + *GpcB1/ Yr36 + Yr15* | 12.80 | 2.0 |
| 50 | PBW550 + *GpcB1/ Yr36 + Yr15* | 13.84 | 1.5 |
| 51 | PBW550 + *GpcB1/ Yr36 + Yr15* | 12.78 | 2.1 |
| 52 | PBW550 + *GpcB1/ Yr36 + Yr15* | 12.94 | 2.5 |
| 53 | PBW550 + *GpcB1/ Yr36 + Yr15* | 12.08 | 1.9 |
| 54 | PBW550 + *GpcB1/ Yr36 + Yr15* | 12.17 | 2.5 |
| 55 | PBW550 + *GpcB1/ Yr36 + Yr15* | 13.23 | 2.3 |
| 56 | PBW550 + *GpcB1/ Yr36 + Yr15* | 12.39 | 2.4 |
| 57 | PBW550 + *GpcB1/ Yr36 + Yr15* | - | 1.7 |
| 58 | PBW550 + *GpcB1/ Yr36 + Yr15* | - | 1.9 |
| 59 | PBW550 + *GpcB1/ Yr36 + Yr15* | - | - |
| 60 | PBW550 + *GpcB1/ Yr36 + Yr15* | 12.54 | 2.1 |
| 61 | PBW550 + *GpcB1/ Yr36 + Yr15* | 13.92 | 3.5 |
| 62 | PBW550 + *GpcB1/ Yr36 + Yr15* | 12.66 | 3.7 |
| 63 | PBW550 + *GpcB1/ Yr36 + Yr15* | - | 2.0 |
| 64 | PBW550 + *GpcB1/ Yr36 + Yr15* | 12.42 | 2.4 |
| 65 | PBW550 + *GpcB1/ Yr36 + Yr15* | 13.51 | 2.2 |
| 66 | PBW550 + *GpcB1/ Yr36 + Yr15* | 12.70 | 2.0 |
| 67 | PBW550 + *GpcB1/ Yr36 + Yr15* | 14.06 | 2.1 |
| 68 | PBW550 + *GpcB1/ Yr36 + Yr15* | 13.41 | 2.5 |
| 69 | PBW550 + *GpcB1/ Yr36 + Yr15* | 13.07 | 2.3 |
| 70 | PBW550 + *GpcB1/ Yr36 + Yr15* | 13.29 | 1.5 |
| 71 | PBW550 + *GpcB1/ Yr36 + Yr15* | 13.91 | 1.7 |
| 72 | PBW550 + *GpcB1/ Yr36 + Yr15* | 13.11 | 0.9 |
| 73 | PBW550 + *GpcB1/ Yr36 + Yr15* | 13.32 | 1.1 |
| 74 | PBW550 + *GpcB1/ Yr36 + Yr15* | 12.16 | 1.7 |
| 75 | PBW550 + *GpcB1/ Yr36 + Yr15* | 12.84 | 2.0 |
| 76 | PBW550 + *GpcB1/ Yr36 + Yr15* | 12.73 | 2.3 |
| 77 | PBW550 + *GpcB1/ Yr36 + Yr15* | 12.41 | 1.9 |
| 78 | PBW550 + *GpcB1/ Yr36 + Yr15* | 13.07 | 2.1 |
| 79 | PBW550 + *GpcB1/ Yr36 + Yr15* | 12.43 | 1.7 |
| 80 | 09014/23 (C) | 12.48 | 1.9 |
| 81 | BWL 6209 (C) | 12.57 | 3.9 |

**S3: Carotenoid content (ppm) of F_2_ plants of cross BC_1_F_5_ 18/ F_6_ 3**

| **Sr. No.** | **Carotenoid (PPM)** | **Sr. No.** | **Carotenoid (PPM)** | **Sr. No.** | **Carotenoid (PPM)** | **Sr. No.** | **Carotenoid (PPM)** | **Sr. No.** | **Carotenoid (PPM)** |
| --- | --- | --- | --- | --- | --- | --- | --- | --- | --- |
| 1 | 1.60 | 6 | 2.50 | 11 | 2.87 | 16 | 2.76 | 21 | 3.74 |
| 2 | 0.68 | 7 | 2.99 | 12 | 2.21 | 17 | 1.84 | 22 | 1.91 |
| 3 | 0.47 | 8 | 1.84 | 13 | 4.14 | 18 | 0.61 | - | - |
| 4 | 2.71 | 9 | 2.45 | 14 | 1.13 | 19 | 2.50 | - | - |
| 5 | 1.34 | 10 | 2.35 | 15 | 2.12 | 20 | 0.75 | - | - |

**S4: Carotene content (ppm) of F_2_ plants of cross BC_1_F_5_ 154/ F_6_ 3**

| **Sr. No.** | **Carotenoid (PPM)** |
| --- | --- |
| 1 | 3.44 |
| **2** | **8.03** |
| 3 | 4.33 |
| 4 | 3.86 |
| **5** | **8.43** |
| **6** | **10.08** |
| **7** | **8.01** |
| **8** | **12.41** |
| **9** | **8.95** |
| **10** | **10.62** |
| **11** | **7.7** |
| 12 | 3.63 |
| 13 | 3.44 |
| **14** | **11.04** |
| **15** | **11.75** |
| **16** | **7.32** |
| **17** | **6.4** |
| 18 | 3.44 |
| **19** | **7.49** |
| 20 | 5.58 |
| 21 | 3.67 |
| 22 | 4.4 |
| **23** | **6.1** |
| **24** | **8.83** |
| 25 | 3.89 |
| **26** | **7.84** |
| **27** | **7.51** |
| 28 | 4.87 |
| 29 | 5.7 |
| 30 | 3.65 |
| **31** | **7.51** |
| 32 | 2.19 |
| 33 | 5.67 |
| **34** | **8.45** |
| **35** | **7.79** |
| 36 | 5.42 |
| **37** | **12.83** |
| 38 | 3.81 |
| 39 | - |
| 40 | 2.78 |
| **41** | **6.97** |
| **42** | **14.06** |
| **43** | **7.28** |
| 44 | 5.58 |
| **45** | **8.48** |
| **46** | **7.28** |
| 47 | 5.93 |
| **48** | **7.35** |
| **49** | **6.76** |
| 50 | 5.49 |
| 51 | 5.65 |
| 52 | 2.9 |
| 53 | 5.84 |
| **54** | **7.58** |
| **55** | **12.2** |
| 56 | 5.09 |
| **57** | **9.7** |
| **58** | **10.55** |
| **59** | **6.78** |
| 60 | - |
| **61** | **7.42** |
| 62 | 5.84 |
| **63** | **6.5** |
| **64** | **7.46** |
| 65 | 4.83 |
| 66 | 3.53 |
| **67** | **6.88** |
| 68 | - |
| 69 | 5.23 |
| **70** | **6.64** |
| 71 | 2.45 |
| **72** | **7.77** |
| 73 | - |
| 74 | 4 |
| 75 | - |
| 76 | 4.52 |
| **77** | **11.68** |
| 78 | 3.63 |
| **79** | **6.24** |
| **80** | **11.35** |
| **81** | **11.21** |
| 82 | 3.06 |
| 83 | 5.51 |
| **84** | **8.99** |
| 85 | 3.6 |
| 86 | - |
| **87** | **7.54** |
| **88** | **7.02** |
| **89** | **7.49** |
| 90 | 5.32 |
| **91** | **8.71** |
| 92 | 4.17 |
| 93 | 3.63 |
| 94 | 4.94 |
| 95 | 4.73 |
| **96** | **8.62** |
| 97 | 5.93 |
| 98 | - |
| **99** | **7.02** |
| **100** | **7.68** |
| 101 | 2.76 |
| **102** | **6.62** |
| 103 | 4.03 |
| **104** | **12.17** |
| **105** | **6.22** |
| **106** | **8.38** |
| **107** | **9.16** |
| **108** | **8.45** |
| 109 | - |
| **110** | **6.29** |
| **111** | **6.95** |
| **112** | **6.99** |
| 113 | 5.91 |
| 114 | 3.18 |
| **115** | **12.08** |
| **116** | **6.05** |
| 117 | 4.29 |
| 118 | 5.58 |
| **119** | **6.95** |
| **120** | **6.48** |
| 121 | 2.66 |
| 122 | 5.75 |
| 123 | - |
| 124 | - |
| 125 | - |
| **126** | **8.24** |
| **127** | **7.82** |
| **128** | **7.42** |
| **129** | **6.55** |
| 130 | - |
| **131** | **6.85** |
| **132** | **7.3** |
| **133** | **9.02** |
| 134 | 3.11 |
| **135** | **7.3** |
| **136** | **6.45** |
| **137** | **11.09** |
| 138 | 2.68 |
| 139 | 3.91 |
| 140 | 4.76 |
| **141** | **6.73** |
| **142** | **7.28** |
| 143 | - |
| 144 | - |
| 145 | 4.78 |
| 146 | 3.44 |
| **147** | **11** |
| 148 | - |
| 149 | 5.6 |
| **150** | **8.12** |
| 151 | 5.89 |
| 152 | - |
| 153 | 5.86 |
| **154** | **6.08** |
| 155 | 5.39 |
| **156** | **6.17** |
| 157 | - |
| 158 | - |
| 159 | 5.65 |
| **160** | **8.41** |
| 161 | 5.77 |
| **162** | **9.72** |
| 163 | 5.35 |
| 164 | 3.34 |
| 165 | 2.94 |
| **166** | **6.88** |
| **167** | **10.03** |
| 168 | 1.58 |
| 169 | 4.78 |
| **170** | **9.16** |
| 171 | 5.98 |
| 172 | 5.16 |
| **173** | **6.95** |
| 174 | - |
| **175** | **7.02** |
| **176** | **6.03** |
| **177** | **7.46** |

**S5: Carotenoid content (ppm) of F_2_ plants of cross BC_1_F_5_ 23/ F_6_ 3**

| **Sr. No.** | **Carotenoid (PPM)** |
| --- | --- |
| 1 | 1.55 |
| 2 | 2.50 |
| 3 | 1.39 |
| 4 | 3.34 |
| 5 | 1.15 |
| 6 | 0.89 |
| 7 | 0.80 |
| 8 | 3.70 |
| 9 | 1.13 |
| 10 | 2.99 |
| 11 | 2.50 |
| 12 | 1.51 |
| 13 | 1.08 |
| 14 | 2.90 |
| 15 | 2.52 |
| 16 | 2.54 |
| 17 | 1.91 |
| 18 | 0.68 |
| 19 | 2.12 |
| 20 | 1.30 |
| 21 | 1.86 |
| 22 | 2.94 |
| 23 | 1.79 |
| 24 | 1.25 |
| 25 | 1.67 |
| 26 | 1.84 |
| 27 | - |
| 28 | 1.13 |
| 29 | 1.86 |
| 30 | 0.71 |
| 31 | 1.65 |
| 32 | 2.76 |
| 33 | 2.52 |
| 34 | - |
| 35 | 1.58 |
| 36 | - |
| 37 | 1.44 |
| 38 | 2.35 |
| 39 | 2.05 |
| 40 | 0.68 |
| 41 | 1.20 |
| 42 | - |
| 43 | 2.45 |
| 44 | 1.98 |
| 45 | 1.32 |
| 46 | 1.27 |
| 47 | 2.90 |
| 48 | 0.61 |
| 49 | 2.64 |
| 50 | 2.78 |
| 51 | 1.25 |
| 52 | 0.97 |
| 53 | 0.94 |
| 54 | 2.21 |
| 55 | 2.33 |
| 56 | 2.76 |
| 57 | - |
| 58 | 1.44 |
| 59 | 0.68 |
| 60 | 2.35 |
| 61 | 0.87 |
| 62 | 1.55 |
| 63 | 2.45 |
| 64 | 2.28 |
| 65 | 1.32 |
| 66 | 1.01 |
| 67 | 2.12 |
| 68 | 1.48 |
| 69 | 2.57 |
| 70 | 1.34 |
| 71 | 2.19 |
| 72 | 0.71 |
| 73 | 2.26 |
| 74 | 1.53 |
| 75 | 2.10 |
| 76 | 0.80 |
| 77 | 1.53 |
| 78 | 3.70 |
| 79 | 1.32 |
| 80 | 0.64 |
| 81 | - |
| 82 | 0.75 |
| 83 | 2.61 |
| 84 | 1.46 |
| 85 | 1.08 |
| 86 | 3.74 |
| 87 | 0.71 |
| 88 | 3.77 |
| 89 | 1.95 |
| 90 | 1.41 |
| 91 | 1.06 |
| 92 | 2.26 |
| 93 | - |
| 94 | 2.83 |
| 95 | 3.37 |
| 96 | 1.01 |
| 97 | 1.18 |
| 98 | 0.97 |
| 99 | 1.60 |
| 100 | 3.46 |
| 101 | 1.37 |
| 102 | 1.74 |
| 103 | 1.22 |
| 104 | 0.82 |
| 105 | 2.87 |
| 106 | 2.21 |
| 107 | 1.41 |
| 108 | 1.53 |
| 109 | 1.51 |
| 110 | 1.06 |
| 111 | 1.86 |
| 112 | 1.55 |
| 113 | 0.94 |
| 114 | 2.47 |
| 115 | 1.30 |
| 116 | 3.11 |
| 117 | 3.32 |
| 118 | 1.01 |
| 119 | 1.39 |
| 120 | 1.25 |
| 121 | 1.77 |
| 122 | 1.79 |
| 123 | 1.44 |
| 124 | 1.48 |
| 125 | - |
| 126 | 1.39 |
| 127 | 2.26 |
| 128 | 1.62 |
| 129 | 2.64 |
| 130 | 1.34 |
| 131 | 4.26 |
| 132 | 2.97 |
| 133 | 1.46 |
| 134 | 1.08 |
| 135 | 1.44 |
| 136 | 0.52 |
| 137 | 0.78 |
| 138 | 0.99 |
| 139 | 1.20 |
| 140 | 2.12 |
| 141 | 3.86 |
| 142 | 1.74 |
| 143 | 3.39 |
| 144 | 1.60 |
| 145 | 1.22 |
| 146 | 1.41 |
| 147 | 2.40 |
| 148 | 1.04 |
| 149 | 1.34 |
| 150 | 2.31 |
| 151 | 1.70 |
| 152 | 1.72 |
| 153 | 2.85 |
| 154 | 2.64 |
| 155 | 2.21 |
| 156 | 1.08 |
| 157 | 0.92 |
| 158 | 2.33 |
| 159 | 1.62 |
| 160 | 0.75 |
| 161 | 3.67 |
| 162 | 1.79 |
| 163 | 1.27 |
| 164 | 0.78 |
| 165 | 3.51 |
| 166 | 3.16 |
| 167 | 2.35 |
| 168 | 2.64 |
| 169 | 0.73 |
| 170 | 1.39 |
| 171 | 2.76 |
| 172 | 1.51 |
| 173 | 2.57 |
| 174 | 2.47 |
| 175 | 1.65 |
| 176 | 1.51 |
| 177 | 2.85 |
| 178 | 1.79 |
| 179 | 2.90 |
| 180 | 1.79 |
| 181 | 3.11 |
| 182 | 0.45 |
| 183 | 2.90 |
| 184 | 1.84 |
| 185 | 2.87 |
| 186 | 4.12 |
| 187 | 2.12 |
| 188 | 1.27 |
| 189 | 3.23 |
| 190 | - |
| 191 | 2.73 |
| 192 | 2.76 |
| 193 | 1.81 |
| 194 | 3.27 |
| 195 | 4.31 |
| 196 | 4.29 |
| 197 | 1.91 |
| 198 | 6.55 |
| 199 | 3.32 |
| 200 | 1.15 |
| 201 | 1.06 |
| 202 | - |
| 203 | 2.57 |
| 204 | 3.23 |
| 205 | 3.79 |
| 206 | 3.74 |
| 207 | 1.53 |
| 208 | 0.85 |
| 209 | 1.65 |
| 210 | 0.80 |
| 211 | 2.03 |
| 212 | 5.93 |
| 213 | 2.12 |
| 214 | 3.01 |
| 215 | 1.13 |
| 216 | 0.66 |
| 217 | 2.47 |
| 218 | 1.11 |

**S6: Carotenoid content (ppm) of F_2_ plants of cross BC_1_F_5_ 20/ F_6_ 3**

| **Sr. No.** | **Carotenoid (PPM)** |
| --- | --- |
| 1 | 2.73 |
| 2 | 1.48 |
| 3 | 2.10 |
| 4 | 2.87 |
| 5 | 3.20 |
| 6 | 4.45 |
| 7 | 3.11 |
| 8 | 3.86 |
| 9 | 2.00 |
| 10 | 3.81 |
| 11 | 2.64 |
| 12 | 2.85 |
| 13 | 1.62 |
| 14 | 1.18 |
| 15 | 1.51 |
| 16 | 2.50 |
| 17 | 1.72 |
| 18 | 3.48 |
| 19 | 1.60 |
| 20 | 2.68 |
| 21 | 1.41 |
| 22 | 4.00 |
| 23 | 3.81 |
| 24 | 2.26 |
| 25 | 2.92 |
| 26 | 3.11 |
| 27 | 3.89 |
| 28 | 2.71 |
| 29 | 2.19 |
| 30 | 2.50 |
| 31 | 3.16 |
| 32 | 1.58 |
| 33 | 2.14 |
| 34 | 1.86 |
| 35 | 3.18 |
| 36 | 1.88 |
| 37 | 3.86 |
| 38 | - |
| 39 | 1.34 |
| 40 | 2.59 |
| 41 | 3.63 |
| 42 | 4.07 |
| 43 | 1.04 |
| 44 | 1.72 |
| 45 | 4.10 |
| 46 | 4.07 |
| 47 | 1.86 |
| 48 | - |
| 49 | 3.70 |
| 50 | 4.03 |
| 51 | - |
| 52 | 1.18 |
| 53 | 9.49 |
| 54 | 3.39 |
| 55 | 3.58 |
| 56 | - |
| 57 | - |
| 58 | - |
| 59 | 1.22 |
| 60 | 2.26 |
| 61 | 3.89 |
| 62 | - |
| 63 | 2.35 |
| 64 | 2.31 |
| 65 | 2.38 |
| 66 | 3.65 |
| 67 | 1.62 |
| 68 | 3.70 |
| 69 | 0.89 |
| 70 | 1.88 |
| 71 | 2.94 |
| 72 | 2.50 |
| 73 | 3.04 |
| 74 | 2.87 |
| 75 | - |
| 76 | 2.73 |
| 77 | 1.88 |
| 78 | 1.58 |
| 79 | 0.92 |
| 80 | 3.84 |
| 81 | 2.61 |
| 82 | 2.28 |
| 83 | - |
| 84 | 3.39 |
| 85 | - |
| 86 | 4.03 |
| 87 | 1.27 |
| 88 | 1.20 |
| 89 | 0.94 |
| 90 | 3.70 |
| 91 | 3.27 |
| 92 | 3.18 |
| 93 | 4.92 |
| 94 | 2.66 |
| 95 | 1.60 |
| 96 | 1.72 |
| 97 | - |
| 98 | 4.57 |
| 99 | 2.10 |
| 100 | 2.28 |
| 101 | 1.93 |
| 102 | 1.34 |
| 103 | 2.05 |
| 104 | 1.20 |
| 105 | 3.37 |
| 106 | 4.43 |
| 107 | - |
| 108 | 5.82 |
| 109 | 2.71 |
| 110 | 2.68 |
| 111 | 2.28 |
| 112 | 3.37 |
| 113 | 3.23 |
| 114 | 5.44 |
| 115 | 2.71 |
| 116 | - |
| 117 | 2.80 |
| 118 | 2.12 |
| 119 | 1.32 |
| 120 | 4.29 |
| 121 | 3.84 |
| 122 | 1.93 |
| 123 | 1.22 |
| 124 | 3.46 |
| 125 | 5.51 |
| 126 | 3.84 |
| 127 | 2.64 |
| 128 | 3.58 |
| 129 | 4.87 |
| 130 | 1.91 |
| 131 | 1.34 |
| 132 | 2.50 |
| 133 | 3.96 |
| 134 | 2.54 |
| 135 | 5.67 |
| 136 | 2.40 |
| 137 | 5.89 |
| 138 | 2.17 |
| 139 | 3.27 |
| 140 | 0.71 |
| 141 | 3.32 |
| 142 | 2.87 |
| 143 | 2.61 |
| 144 | 2.05 |
| 145 | 2.68 |
| 146 | 4.21 |
| 147 | 5.53 |
| 148 | 2.07 |
| 149 | 1.11 |
| 150 | 1.48 |
| 151 | 1.91 |
| 152 | 3.18 |
| 153 | 3.30 |
| 154 | 3.48 |
| 155 | 2.28 |
| 156 | 4.57 |
| 157 | 4.69 |
| 158 | 1.48 |
| 159 | - |
| 160 | 1.81 |
| 161 | 1.79 |
| 162 | 3.32 |
| 163 | 5.09 |
| 164 | 1.84 |
| 165 | 1.53 |
| 166 | 4.12 |
| 167 | - |
| 168 | 3.39 |
| 169 | 4.73 |
| 170 | 4.57 |
| 171 | 1.67 |
| 172 | 3.04 |
| 173 | 4.64 |
| 174 | 3.41 |
| 175 | - |
| 176 | 5.04 |
| 177 | 0.59 |
| 178 | 2.87 |
| 179 | 3.27 |
| 180 | 1.88 |
| 181 | - |
| 182 | 1.70 |
| 183 | 1.01 |
| 184 | 1.39 |
| 185 | 2.47 |
| 186 | 2.35 |
| 187 | 2.43 |
| 188 | 3.16 |
| 189 | 1.72 |
| 190 | 4.66 |
| 191 | 1.81 |
| 192 | 2.10 |
| 193 | 1.06 |
| 194 | 2.71 |
| 195 | 1.93 |
| 196 | 2.14 |
| 197 | 1.37 |
| 198 | 1.39 |
| 199 | 0.99 |
| 200 | 1.44 |
| 201 | 4.97 |
| 202 | 3.63 |
| 203 | 1.91 |
| 204 | 1.70 |
| 205 | 2.85 |
| 206 | 2.21 |
| 207 | 4.52 |
| 208 | 2.66 |
| 209 | 2.68 |
| 210 | 4.36 |
| 211 | 4.38 |
| 212 | 3.70 |
| 213 | 2.54 |
| 214 | 3.32 |
| 215 | 1.74 |
| 216 | 3.30 |
| 217 | 2.21 |
| 218 | 1.48 |
| 219 | 1.06 |
| 220 | 3.25 |
| 221 | 2.26 |
| 222 | 4.36 |
| 223 | 2.87 |
| 224 | 3.04 |
| 225 | 3.86 |
| 226 | 2.19 |
| 227 | 2.38 |
| 228 | 2.21 |
| 229 | 4.69 |
| 230 | 2.38 |
| 231 | 2.47 |
| 232 | 2.07 |

**Table S7: Mean, SD, SE, Median, Maximum, Minimum values for carotenoid content in selected F_2_ populations**

| **Sr. No.** | **Cross** | **N** | **Mean** | **SE Mean** | **SD** | **Minimum** | **Maximum** | **Median** |
| --- | --- | --- | --- | --- | --- | --- | --- | --- |
|  | BC_1_F_5_ 18/ F_6_ 3 | 22 | 2.069 | 0.209 | 0.981 | 0.471 | 4.144 | 2.166 |
|  | BC_1_F_5_ 20/ F_6_ 3 | 232 | 2.793 | 0.084 | 1.241 | 0.588 | 9.489 | 2.637 |
|  | BC_1_F_5_ 23/ F_6_ 3 | 218 | 1.966 | 0.068 | 0.987 | 0.447 | 6.546 | 1.742 |
|  | BC_1_F_5_ 154/ F_6_ 3 | 177 | 6.579 | 0.197 | 2.478 | 1.578 | 14.058 | 6.464 |
|  | Checks | 19 | 2.314 | 0.189 | 0.803 | 0..871 | 3.461 | 2.402 |

**Table S8: Selected 89 lines on the basis of carotenoid content**

| **Sr. No.** | **Plant** | **Carotenoid (ppm)** | **PPO (out of 10)** |
| --- | --- | --- | --- |
| 1 | 2 | 8.03 | 3.8 |
| 2 | 5 | 8.43 | 3.2 |
| 3 | 6 | 10.08 | 3.5 |
| 4 | 7 | 8.01 | 4.5 |
| 5 | 8 | 12.41 | 3.4 |
| 6 | 9 | 8.95 | 3.3 |
| **7** | **10** | **10.62** | **2.6** |
| 8 | 11 | 7.7 | 3.8 |
| 9 | 14 | 11.04 | 3 |
| **10** | **15** | **11.75** | **2.6** |
| 11 | 16 | 7.32 | 3.4 |
| 12 | 17 | 6.4 | 3.2 |
| 13 | 19 | 7.49 | 3.8 |
| 14 | 23 | 6.1 | 3.2 |
| **15** | **24** | **8.83** | **1.4** |
| 16 | 26 | 7.84 | 3.6 |
| **17** | **27** | **7.51** | **2.8** |
| **18** | **31** | **7.51** | **1.4** |
| 19 | 34 | 8.45 | 3.4 |
| 20 | 35 | 7.79 | 3.3 |
| 21 | 37 | 12.83 | 3.8 |
| 22 | 41 | 6.97 | 3.2 |
| 23 | 42 | 14.06 | 3.2 |
| **24** | **43** | **7.28** | **2** |
| 25 | 45 | 8.48 | 4 |
| 26 | 46 | 7.28 | 3.4 |
| 27 | 48 | 7.35 | 3.2 |
| 28 | 49 | 6.76 | 3.2 |
| 29 | 54 | 7.58 | 3.4 |
| 30 | 55 | 12.2 | 3.8 |
| 31 | 57 | 9.7 | 3.4 |
| 32 | 58 | 10.55 | 4.2 |
| 33 | 59 | 6.78 | 3.4 |
| 34 | 61 | 7.42 | 3.6 |
| 35 | 63 | 6.5 | 2.9 |
| **36** | **64** | **7.46** | **2.8** |
| 37 | 67 | 6.88 | 3.5 |
| 38 | 70 | 6.64 | 3.4 |
| 39 | 72 | 7.77 | 3.4 |
| 40 | 77 | 11.68 | 3.6 |
| 41 | 79 | 6.24 | 3.4 |
| 42 | 80 | 11.35 | 3.7 |
| 43 | 81 | 11.21 | 4.6 |
| 44 | 84 | 8.99 | 3.2 |
| **45** | **87** | **7.54** | **2.7** |
| **46** | **88** | **7.02** | **2.4** |
| **47** | **89** | **7.49** | **2.4** |
| 48 | 91 | 8.71 | 3.4 |
| 49 | 96 | 8.62 | 3.6 |
| 50 | 99 | 7.02 | 3.8 |
| 51 | 100 | 7.68 | 3.4 |
| **52** | **102** | **6.62** | **2.2** |
| **53** | **104** | **12.17** | **2.7** |
| 54 | 105 | 6.22 | 3.6 |
| 55 | 106 | 8.38 | 4.8 |
| 56 | 107 | 9.16 | 3.2 |
| **57** | **108** | **8.45** | **2.7** |
| 58 | 110 | 6.29 | 4.4 |
| 59 | 111 | 6.95 | 3.2 |
| 60 | 112 | 6.99 | 3.8 |
| 61 | 115 | 12.08 | 3.6 |
| 62 | 116 | 6.05 | 3.4 |
| 63 | 119 | 6.95 | 3.8 |
| 64 | 120 | 6.48 | 3.6 |
| 65 | 126 | 8.24 | 3.8 |
| 66 | 127 | 7.82 | 3.5 |
| **67** | **128** | **7.42** | **2.8** |
| 68 | 129 | 6.55 | 3.2 |
| 69 | 131 | 6.85 | 3.4 |
| 70 | 132 | 7.3 | 3.4 |
| 71 | 133 | 9.02 | 3.7 |
| 72 | 135 | 7.3 | 3.8 |
| 73 | 136 | 6.45 | 3.4 |
| 74 | 137 | 11.09 | 3.8 |
| 75 | 141 | 6.73 | 3.6 |
| 76 | 142 | 7.28 | 3.2 |
| 77 | 147 | 11 | 3.4 |
| 78 | 150 | 8.12 | 4.5 |
| 79 | 154 | 6.08 | 3 |
| 80 | 156 | 6.17 | 4 |
| **81** | **160** | **8.41** | **2.6** |
| 82 | 162 | 9.72 | 3.8 |
| 83 | 166 | 6.88 | 4 |
| 84 | 167 | 10.03 | 3.8 |
| 85 | 170 | 9.16 | 3.2 |
| 86 | 173 | 6.95 | 3.4 |
| 87 | 175 | 7.02 | 3.8 |
| 88 | 176 | 6.03 | 3.8 |
| 89 | 177 | 7.46 | 3.3 |

**Table S9: Selected 15 lines on the basis of PPO activity in the experiment**

| **Sr. No.** | **Plant** | **Carotenoid (ppm)** | **PPO (//10)** | **Protein content (%)** |
| --- | --- | --- | --- | --- |
| 1 | **10** | **10.62** | **2.6** | **13.5** |
| 2 | 15 | 11.75 | 2.6 | 13.21 |
| 3 | 24 | 8.83 | 1.4 | 12.48 |
| 4 | 27 | 7.51 | 2.8 | 12.87 |
| 5 | **31** | **7.51** | **1.4** | **13.75** |
| 6 | 43 | 7.28 | 2 | 13.42 |
| 7 | 64 | 7.46 | 2.8 | 11.52 |
| 8 | 87 | 7.54 | 2.7 | 14.53 |
| 9 | 88 | 7.02 | 2.4 | 12.56 |
| 10 | 89 | 7.49 | 2.4 | 12.61 |
| 11 | 102 | 6.62 | 2.2 | 12.79 |
| 12 | **104** | **12.17** | **2.7** | **13.74** |
| 13 | 108 | 8.45 | 2.7 | 13.27 |
| 14 | 128 | 7.42 | 2.8 | 10.61 |
| 15 | **160** | **8.41** | **2.6** | **14.06** |

- **Selected lines for further yield trials**
