# Supplementary material for: Combining high carotenoid, grain protein content and rust resistance in wheat for food and nutritional security
Source: Front Genet. 2023 Jan 19;14:1075767. doi: 10.3389/fgene.2023.1075767 (PMC9893017; doi:10.3389/fgene.2023.1075767)
Supplement: Supplementary file 2 [file DataSheet1.docx]

**
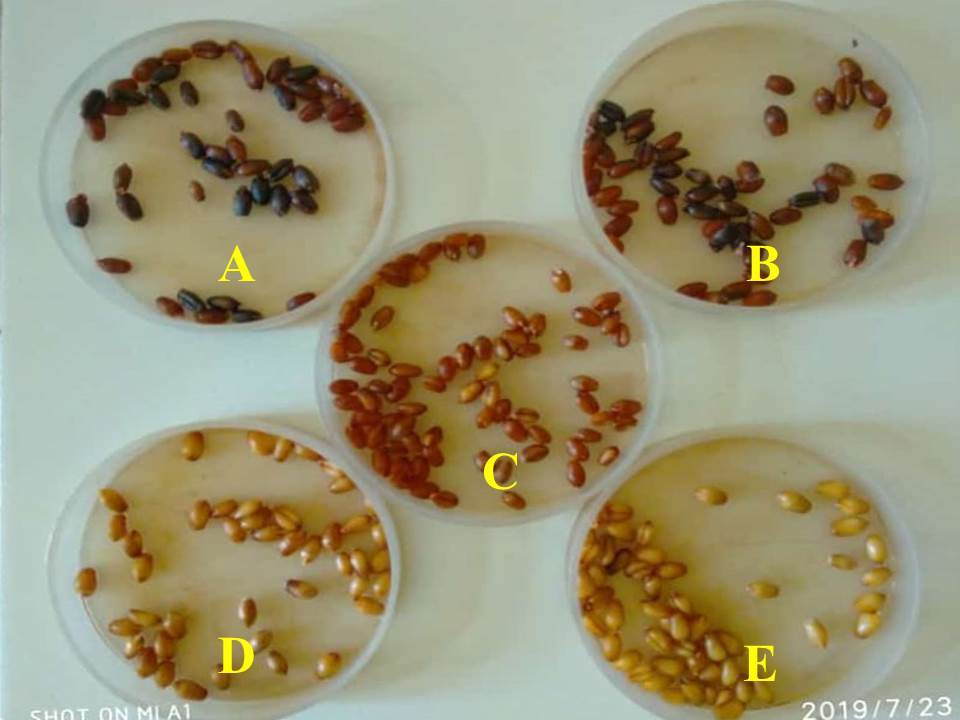
**

**S 1: Phenol reaction and comparison of the genotypes A: HD2977 (High PPO activity), B: line 27 (High PPO), C: Line 2 (Medium PPO), D: Line 3 (Low PPO), E:Line 72 (low PPO)**


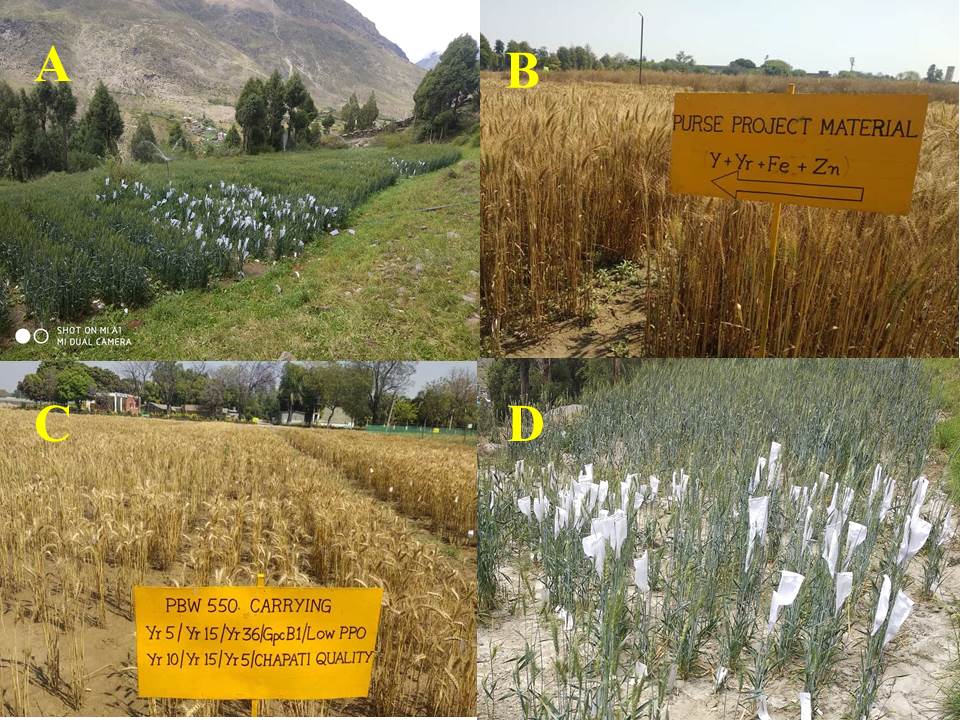


**S2: Field trial of the genotypes in different experiments A: Offseason trial at Keylong (HP), B: trial for Experiment No 1 and 2, C: Trial for Experiment No 3, D: Crossing Block study**
